# Atypical for northern ungulates, energy metabolism is lowest during summer in female wild boars (*Sus scrofa*)

**DOI:** 10.1038/s41598-021-97825-z

**Published:** 2021-09-15

**Authors:** Thomas Ruf, Sebastian G. Vetter, Johanna Painer, Gabrielle Stalder, Claudia Bieber

**Affiliations:** 1 Department of Interdisciplinary Life Sciences, Research Institute of Wildlife Ecology, University of Veterinary Medicine, Savoyenstrasse 1, 1160 Vienna, Austria; 2 Present Address: Department for Farm Animals and Veterinary Public Health, Institute of Animal Welfare Science, University of Veterinary Medicine, Veterinärplatz 1, 1210 Vienna, Austria

**Keywords:** Evolutionary ecology, Metabolism, Ecology, Physiology

## Abstract

Typically, large ungulates show a single seasonal peak of heart rate, a proxy of energy expenditure, in early summer. Different to other large ungulates, wild boar females had peak heart rates early in the year (at ~ April, 1), which likely indicates high costs of reproduction. This peak was followed by a trough over summer and a secondary summit in autumn/early winter, which coincided with the mast seeding of oak trees and the mating season. Wild boars counteracted the effects of cold temperatures by decreasing subcutaneous body temperature by peripheral vasoconstriction. They also passively gained solar radiation energy by basking in the sun. However, the shape of the seasonal rhythm in HR indicates that it was apparently not primarily caused by thermoregulatory costs but by the costs of reproduction. Wild boar farrow early in the year, visible in high HRs and sudden changes in intraperitoneal body temperature of females. Arguably, a prerequisite for this early reproduction as well as for high energy metabolism over winter is the broad variety of food consumed by this species, i.e., the omnivorous lifestyle. Extremely warm and dry summers, as experienced during the study years (2017, 2018), may increasingly become a bottleneck for food intake of wild boar.

## Introduction

“Climate plays an important part in determining the average numbers of a species, and periodical seasons of extreme cold or draught, I believe it to be the most effective of all checks”. With this remark, Darwin^1^ identified the seasonality of environments as an important force in natural selection. Mammals can respond to seasonal environments in different ways. If a shortage of food and times of increased energy demands, e.g., for heat production, coincide they can adapt by reducing energy turnover. This is achieved, for instance, by minimizing locomotor activity, using regional heterothermy, and hypometabolism^2^. Certain, mostly small mammals may even seasonally retreat into burrows or caves and drastically lower energy expenditure by hibernation^3^. Others may meet increased energy demands by increasing food uptake, if environmental conditions permit. Most north-temperate large ungulates choose the first avenue, that is, they downregulate energy expenditure, heat loss, and activity during winter^2,4^. A large mammal whose seasonal responses are difficult to predict, however, is the wild boar (*Sus scrofa*)*.*

The wild boar is one of most widespread species worldwide. Its evolutionary lineage originates from islands (Philippines, Indonesia) in South-East Asia^5^. Interestingly, the oldest cave painting in the world, at least 45.5 ka old, in Sulawesi, is that of a pig^6^. The wild boar has dispersed across Eurasia, was domesticated independently several times, and currently inhabits all continents except Antarctica^5,7^. Thus, the wild boar is one of the species whose geographical range has expanded far beyond its native range^8^. Two important aspects that have facilitated this success are the high fecundity of wild boars and their highly opportunistic diet, which includes many plants and animals^7,9^. In addition, the wild boar is a pulsed resource consumer heavily exploiting the mast seeding of trees, such as acorn and beech seeds, which directly affects reproductive success and demography in this species^10,11^.

Wild boar are seasonal breeders^12^ with conception events occurring from September to March^13^ in central Europe, but, within this range, relatively early in the year under good environmental conditions^14^. The rut in fall/winter involves strong male competition in this polygynous species. Gestation lasts on average 115 days, and most young are born in March and April in central Europe^15^. Although wild boars typically have one litter per year, adult females may have a second litter whenever they fail to raise the first^15^. Compared with other ungulates, the average litter size (~ 5 young) and fecundity of wild boar under favourable environmental conditions is very high, e.g.^15–17^. The onset of female reproduction primarily depends on body weight, and females with a good food supply start to reproduce at an age under a year^16,17^. Hence, while the seasonal reproductive cycle in these short-day breeders is principally governed by photoperiod and melatonin^12,18^ environmental conditions such as climate and food supply have a modulating effect on wild boar reproduction.

Apart from their high fecundity, surprisingly little is known about the physiological adaptations that have enabled wild boars to successfully inhabit diverse, often highly seasonal environments. Given that they evolved in warm habitats, such as the Philippines, wild boars seem to be not well suited to thrive in cold environments. The Suidae are among those mammalian clades that have lost functional UCP1, and hence non-shivering thermogenesis in the brown adipose tissue^19^. It is not even clear which ambient temperatures represent cold stress for this species, as to our knowledge the thermoneutral zone for wild-type adults is not known. However, measurements in Poland suggest that the lower critical temperature of adults is − 6 °C or below, while that of still growing animals is − 3 °C^20,21^. The effects of low temperatures will be locally different, however, because wild boars have a pronounced body size gradient within Europe, with smaller sizes in warmer regions^22^. In models of the geographical distribution, mean annual temperature was the most important factor predicting wild boar presence, and, not surprisingly, the models predicted a higher probability to encounter this species in the warmer areas^8^.

On the other hand, during the last century wild boar ranges have expanded and the species has been detected even in areas where the average snow-depth exceeds 50–70 cm and where the average winter temperatures are below − 30 °C^8^. Wild boars were even recorded close to the Arctic circle^23^. Wild boar juveniles indeed suffer from severe winter conditions^24^, but abundant availability of food resources can outweigh the negative effects of cold winters on population growth^22^. The ability to withstand cold will be aided by the wild boar juveniles capacity for an alternative non-shivering thermogenesis in skeletal muscles^25^. This heat generation mechanism may have been important for the evolution of endothermy^26^ and could have played a crucial role in the dispersion of wild boars. Also, it has long been known that cold-exposed domestic swine shunt blood away from the body surface, thereby forming an insulating outer layer of tissues to minimize heat loss^27^.

To investigate in more detail if and how wild boar physiologically respond to a seasonal environment, we continuously measured heart rate, body temperature (both core and subcutaneous temperatures) as well as locomotor activity in female wild boars equipped with data loggers. Animals were free-ranging in a large (55 ha), wooded outdoor enclosure in Austria, exposed to natural climate. For this study, heart rate serves as a proxy for energy expenditure. Under most circumstances heart rate is correlated with the rate of oxygen consumption and hence the rate of energy expenditure, review in^28^. This approach has been successfully used to quantify seasonal adjustments in other northern ungulates, such as red deer (*Cervus elaphus*)^29^, Przewalski horses (*Equus przewalskii*)^30^, ibex (*Capra ibex*)^31^ or Svalbard reindeer (*Rangifer tarandus*)^32^. Most of these ungulates can inhabit harsh environments and are cold tolerant. For instance, the thermoneutral zone of red deer extends down to − 30.8 °C^33^. However, all of them show a peak energy expenditure in summer, and minimum heart rate in winter. We hypothesized that wild boars, due to their flexible food habits, might differ. In particular, in mast years, when food is abundant, they may not undergo a seasonal rhythm in heart rate at all. We also hypothesized that they would use vasoconstriction to minimize heat loss under decreasing ambient temperatures, and may use basking, similar to Alpine ibex^31^, to minimize the costs for thermoregulation.

## Results

**Figure 1:**
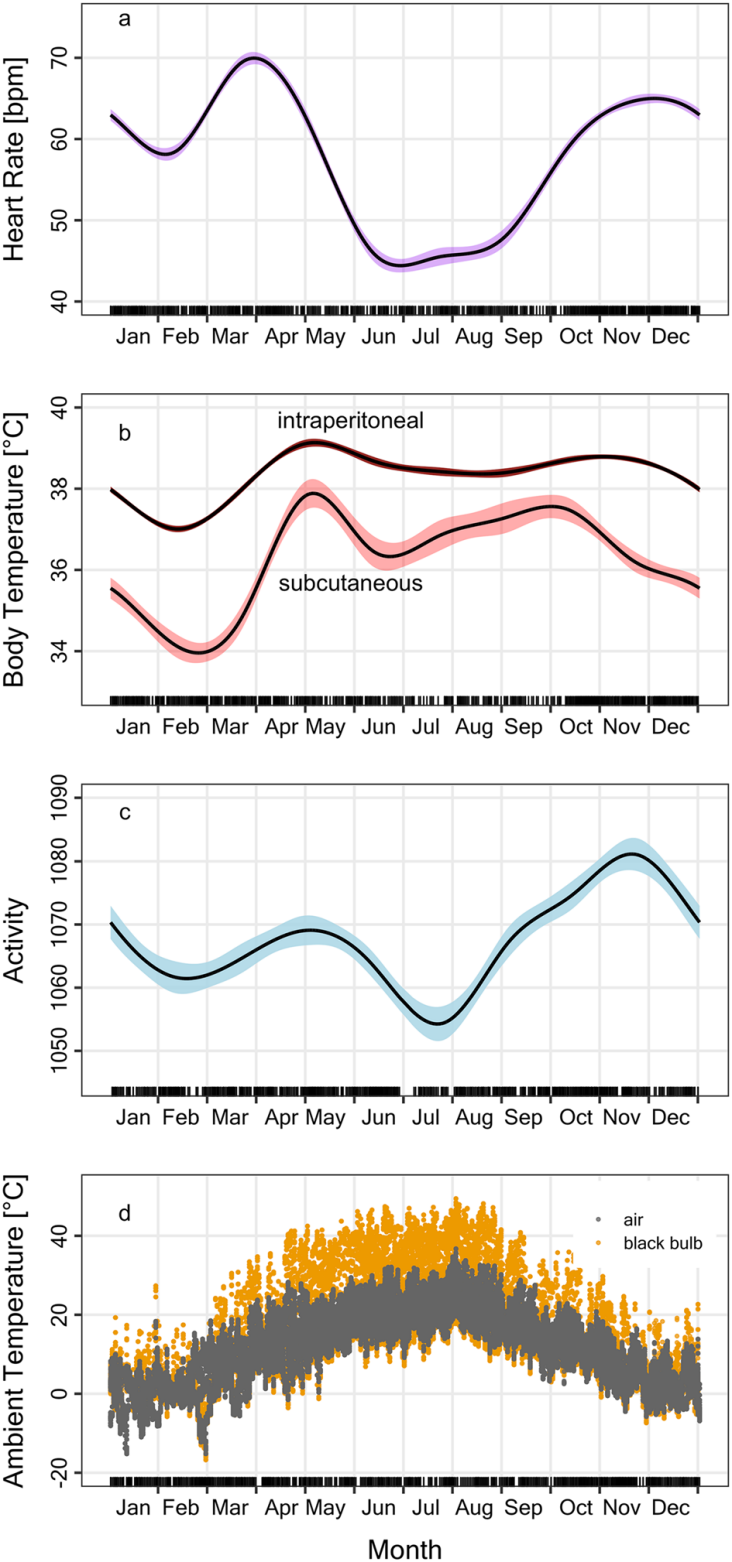
Seasonal time courses of (**a**) heart rate, (**b**) body temperature, (**c**) activity in female wild boars, as well as (**d**) air and black-bulb temperature at the study site. (**a**–**c**) Show predictions from mixed models (GAMMs) that adjusted for individual levels of the ten females. Coloured areas around the lines are 95% confidence intervals. (**d**) Shows temperatures as measured.

Mean hourly HR was significantly affected by all variables investigated (Fig. 1, Table 1). As indicated by the high F-Value, season (DOY) had the strongest effect of all environmental variables (Table 1). Female wild boars showed two peaks of HR, the highest peak around 1 April and a second peak around 1 December, while a trough occurred during the summer months, June–August (Fig. 1a). The seasonal time course of T_bip_ and T_bsc_ was close to parallel to each other, and almost inverse to HR, with highest temperatures during spring to fall (Fig. 1b). The lowest impact on HR had locomotor activity, which peaked in November (Table 1, Fig. 1c). After adjusting for the fluctuation in T_bip_ and T_bsc_, T_a_ as such (Fig. 1d) had only a relatively minor effect on HR (see F-value in Table 1). HR showed a daily rhythm with a clear peak in the evening (at 19:00–20:00 h) both in summer and winter (not shown on graph).

**Table 1:** Results from a GAMM multiple regression model testing for effects on mean hourly heartrate.

Term	Estimate	SE	t-value	P-value
Intercept	77.68	2.81	27.58	< 0.0001

Term		EDF	F-value	P-value
s(DOY)		7.55	73.40	< 0.0001
s(HOUR)		5.88	15.12	< 0.0001
s(T_a_)		5.08	14.53	< 0.0001
s(ACT)		6.84	4.46	< 0.0001
s(T_bip_)		7.66	8.22	< 0.0001
s(T_bsc_)		6.95	38.55	< 0.0001
ID-random		5.76	27.04	< 0.0001

The effective degree of freedom (EDF) is an index of the “wiggliness” of the term-effect. The adjusted R^2^ of the model was 0.349. F-values represent an in index of relative variable importance.

The mean level of HR varied significantly between individuals (Table 1), which was, however, unrelated to their body mass (55.0–90.0 kg, r = − 0.12 for Dec., when HR was available from all ten females).

**Figure 2:**
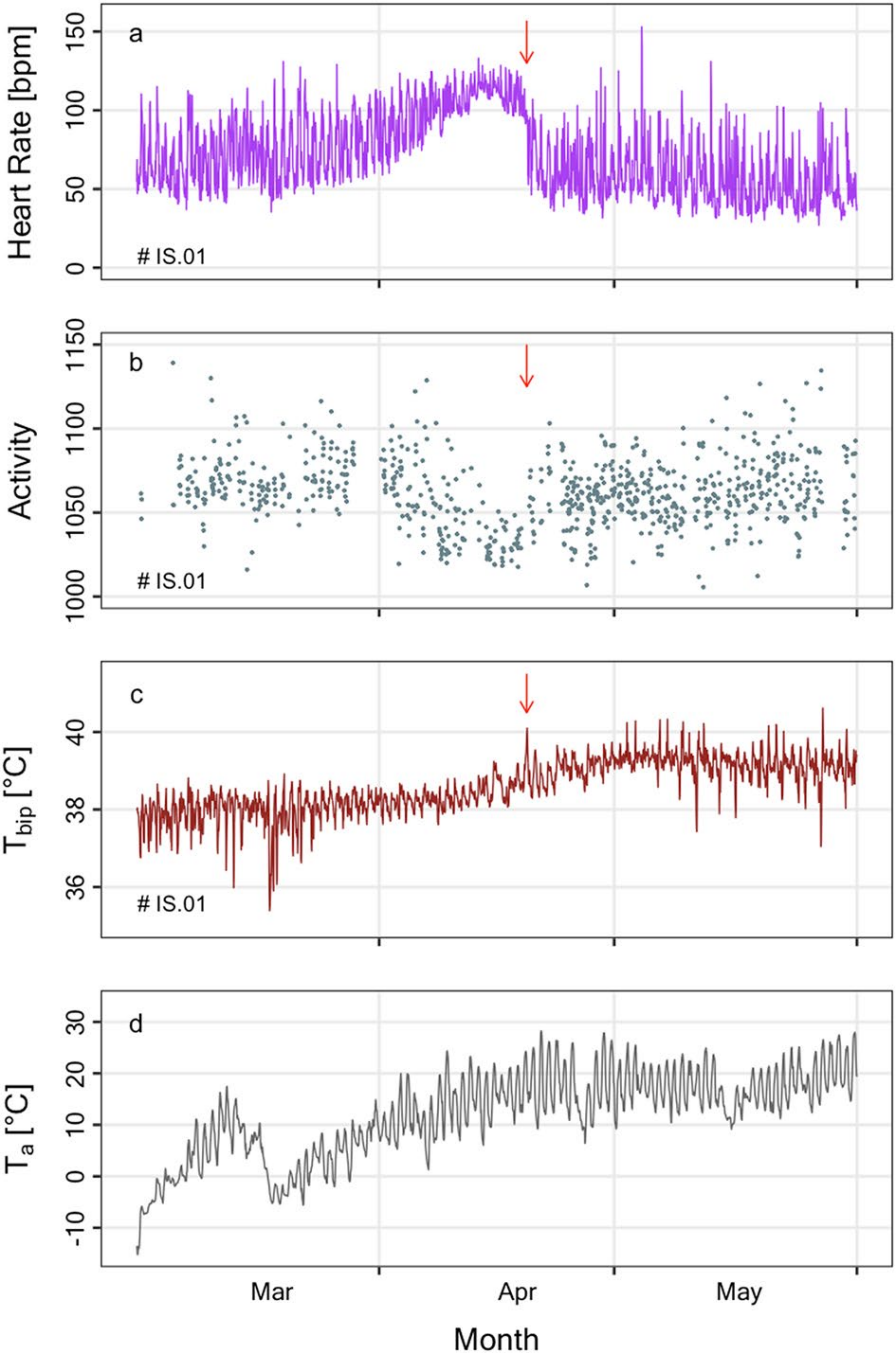
Example for the time courses of hourly means of (**a**) heart rate, (**b**) activity, (**c**) intraperitoneal temperature (T_bip_) and (**d**) ambient temperature (T_a_) early in the year (2018-03-01–2018-05-31). All physiological variables were measured in the same female (IS.01). The red arrows indicate times when the female had a suddenly decreasing HR, resumed activity, and had a spike in body temperature, presumably at parturition.

The spring peak in HR (Fig. 1a) was due to a peculiar pattern of HR in all females equipped with a functioning HR logger at this time of the year (n = 7, female # IS.01 shown as example in Fig. 2). This pattern was characterized by an increase of HR followed by a sudden return to lower levels (Fig. 2a). Female # IS.01 also displayed a lowering of activity in the days around peak HR (Fig. 2b). However, this behaviour could be assessed only in one female, because all others moved outside the activity reception range, presumably into the surrounding woods, at this time of the year. Females further showed a spike in T_bip_ just prior the reduction of HR, and a diminished amplitude of daily fluctuations over several weeks before and after the peak in HR (Fig. 2c). These patterns showed no clear relation to changes in T_a_ in spring (Fig. 2d). We attribute them to gestation, parturition (at a spike in T_bip_ with a sudden change in HR, see red arrows Fig. 2), and the onset of lactation.

**Figure 3:**
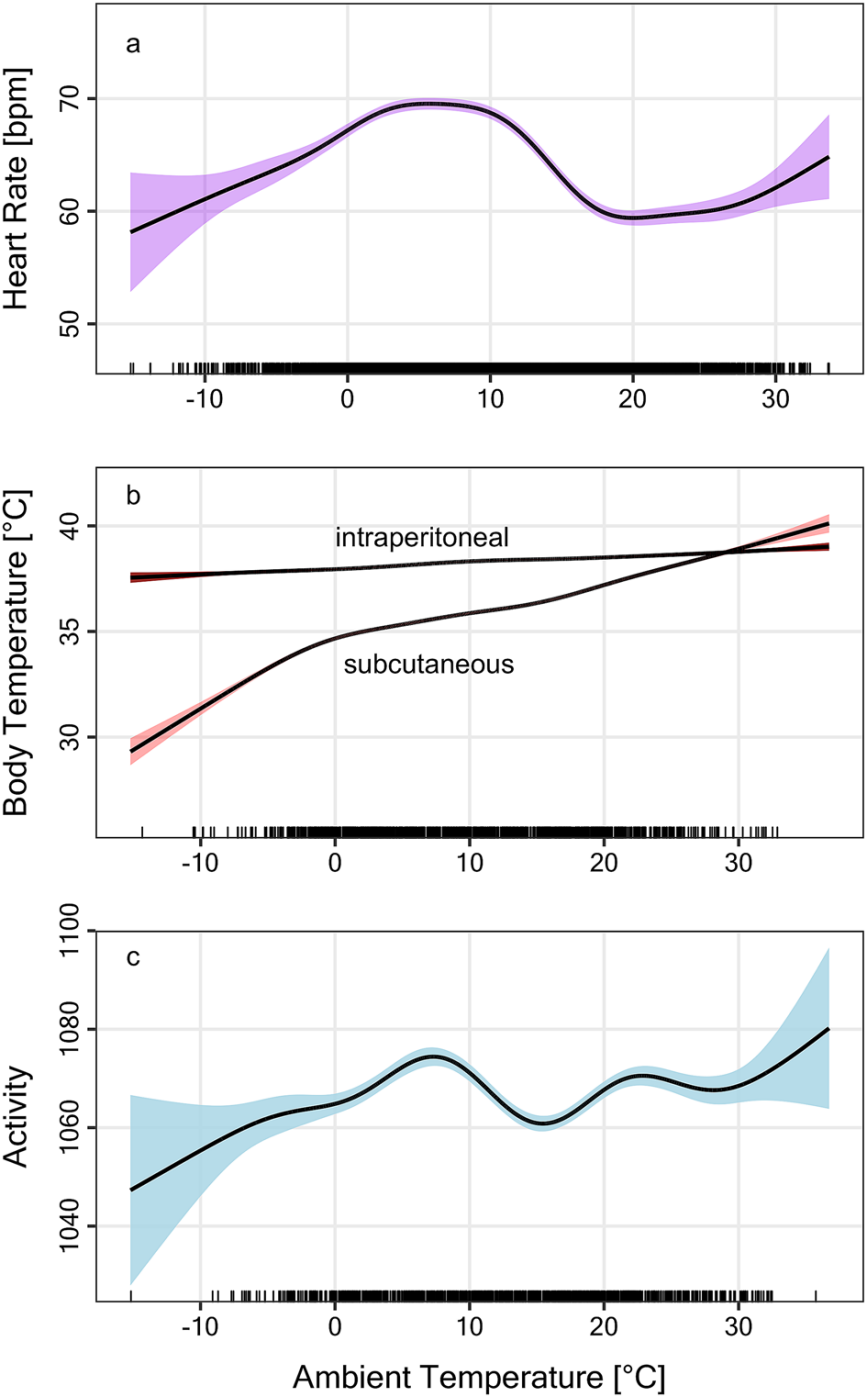
Effects of ambient temperature (T_a_) on (**a**) heart rate, (**b**) body temperature and (**c**) activity in wild boar females. Predictions from mixed models (GAMMs) that adjusted for individual levels of the ten females. Coloured areas around the lines are 95% confidence intervals. HR and activity peaked at ~ + 8 °C (**a**, **c**). As T_a_ decreased both intraperitoneal and subcutaneous T_b_ were lowered, but this decline was much more pronounced in subcutaneous T_b_.

To separate seasonal and T_a_ effects, HR, T_b_ and activity were also analysed as a function of T_a_. T_a_ apparently (after adjusting for the random factor ID) had a moderate impact on all physiological variables (Fig. 3). It affected HR (EDF = 7.89; F = 73.05; P < 0.0001) with elevated HR in a temperature range of ~ 0–12 °C. Note, however, that the amplitude of HR excursions induced by T_a_ (~ 60–70 bpm; Fig. 3a) was less than half the amplitude of seasonal changes in mean HR (~ 45–70 bpm; Fig. 1a). T_bip_ dropped by ~ 1 °C as T_a_ decreased from ~ + 30 to − 10 °C (EDF = 6.20; F = 78.37; P < 0.0001), while the reduction of T_bsc_ was much more pronounced (Fig. 3b; EDF = 8.43; F = 418.9; P < 0.0001). The activity of the animals was also affected by T_a_ (EDF = 7.92; F = 13.34; P < 0.0001), and wild boars tended to become less active at the coldest temperatures recorded (Fig. 3c).

**Figure 4:**
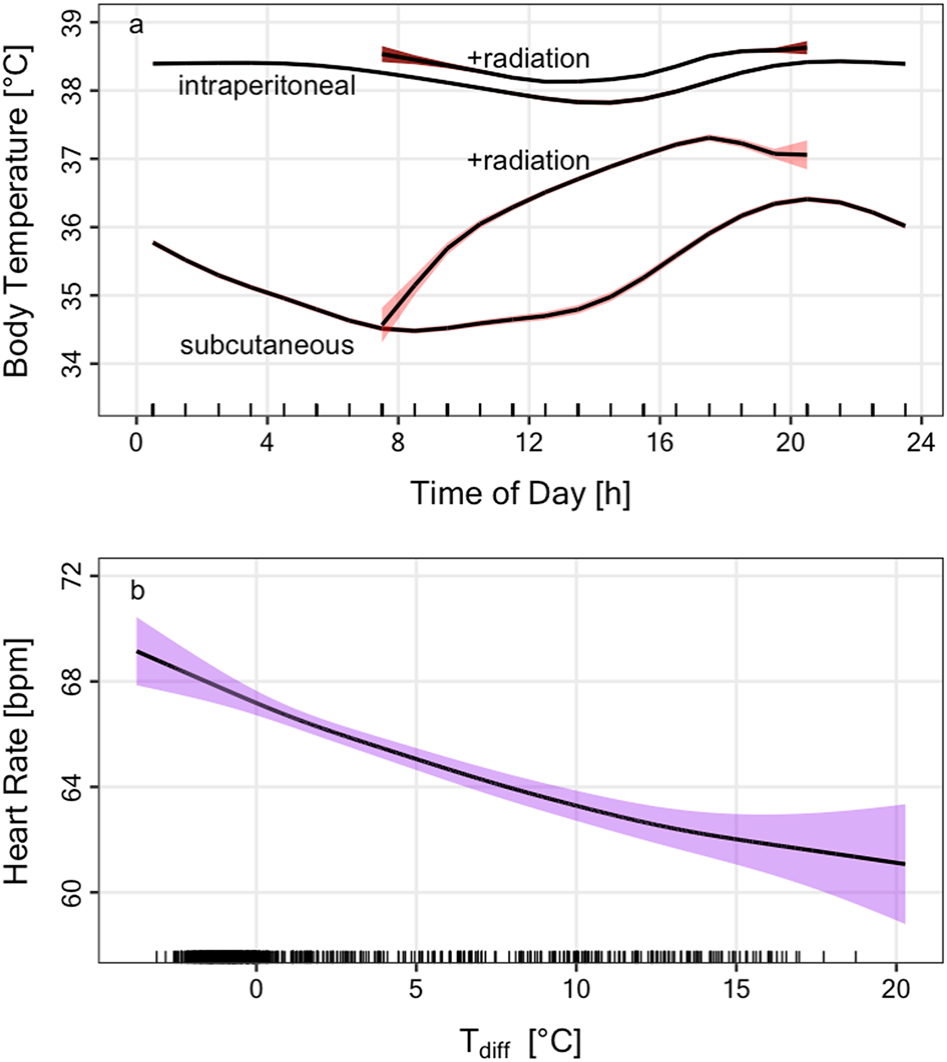
Basking in wild boars. (**a**) Daily time course of intraperitoneal body temperature (T_ip_) and subcutaneous body temperature T_sc_ for times at which solar radiation was low or high (+ radiation). Radiation was considered high when black bulb temperature was above the median, i.e., more than + 5 °C above air temperature. Radiation facilitated re-warming of the body shell and was associated with lower HR. (**b**) Effect of solar radiation (back bulb temperature (T_abb_)—ambient temperature (T_a_)) on HR in wild boars. Predictions from a GAMM model simultaneously adjusting for T_a_. Coloured areas in both panels indicate 95% confidence intervals.

When the difference between black-bulb and air temperature (T_diff_) was considerable (exceeding the median of ~ + 5 °C) this led to a faster daily increase in T_bsc_ and to a slight elevation of T_bip_ (Fig. 4a). After adjusting for the simultaneous effects of hour of day and T_a_ as such, these effects of T_diff_ on T_bip_ (EDF = 3.57; F = 8.16; P < 0.0001) and T_bsc_ (EDF = 5.37; F = 25.92; P < 0.0001) were significant. This uptake of external heat saved energy. For example, when T_diff_ exceeded a threshold of 5 °C during daylight hours (Fig. 4a) the total mean HR was 58.06 ± 0.14 bpm, which is 10% lower than with little or no radiation (T_diff_ < 5 °C; 64.23 ± 0.15 bpm). In fact, with increasing radiation (T_diff_) wild boars had continually lower HR (EDF = 2.366; F = 22.29; P < 0.0001) after adjusting for the effect of T_a_ as such (Fig. 4b).

## Discussion

This is, to our knowledge, the first time that seasonal changes in HR, T_b_ and activity were simultaneously recorded in wild boars. Season had clearly the most pronounced impact on HR, beyond the pure effect of T_a_ (Table 1). Seasonal fluctuations in HR, a proxy for energy expenditure were pronounced, as is typical for northern ungulates, review in^2^. There are three main reasons for mammals to temporarily adjust their energy expenditure: food availability, thermoregulation, and the cost of reproduction. We will discuss the case of the wild boar considering these aspects in turn.

**Figure 5:**
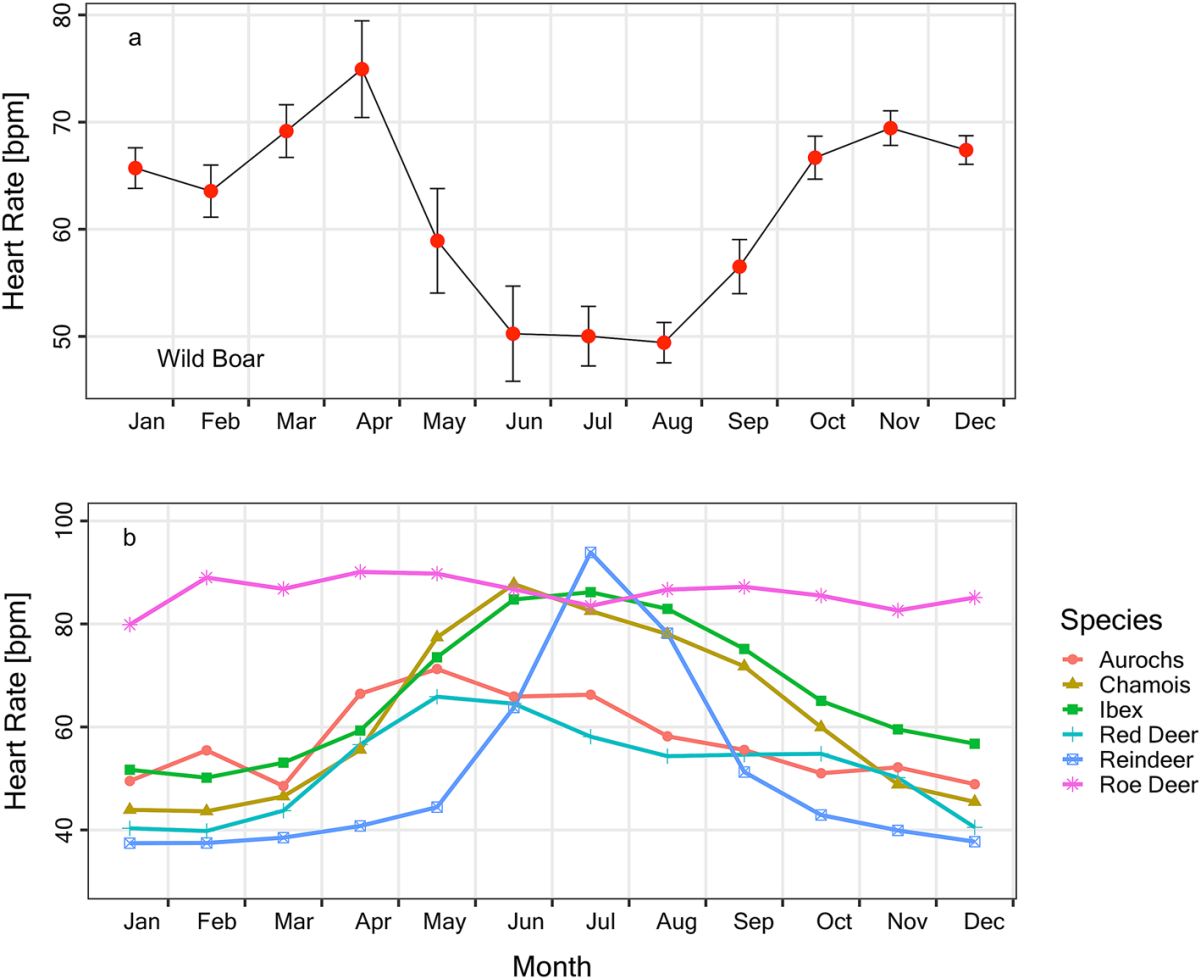
Seasonal time course of heart rate in northern ungulates. (**a**) Mean monthly HR ± SEM in wild boars (present study). (**b**) Traces of mean monthly HR in six species of ruminants. Data in (**b**) from Arnold^2^.

The wild boar is an omnivore consuming mast (such as acorns, beechnuts, chestnuts), roots, green plant matter and agricultural crops as well as animal foods, namely insects, earthworms, birds and mammals^9,34^. This contrasts with most north-temperate ungulates which mostly feed on grass and roughage or are intermediate foragers, with the exception of so called concentrate selectors, review in^35^. In particular, for species that are located toward the grazer spectrum of foraging types, the availability and quality of plant material is considerably lower outside the vegetation season. We suggest that this difference in food selection largely explains the time course of HR in wild boar females, which clearly differs from known seasonal patterns in other ungulates (Fig. 5). Energy expenditure was high in winter and reached a minimum in summer, while the opposite was the case in other large ungulates. Only the roe deer (*Capreolus capreolus*), which is a pronounced concentrate selector, showed a comparatively weak, but detectable seasonal rhythm of energy metabolism cf.^36^. A summer peak in HR is not limited to ruminants (Fig. 5) but also occurs in a monogastric species, the Przewalski horse^37^. Very short seasonal peaks of HR, like in Svalbard reindeer, are thought to reflect the extremely short vegetation season in their habitat^32^. In wild boars, on the other hand, high energy expenditure can be sustained mainly by the availability of energy-rich seeds, which are mass-produced by trees in mast years and available on the ground throughout winter. In mast years, consumption of acorns and beechnuts starts in autumn and continues into spring^9^. Wild boars can even gain body mass over winters following mast years^15^. At our study site an abundant seed producer is the Turkey oak (*Quercus cerris*), and at least a fraction of oaks was seeding in each study year. The seed production by Turkey oaks is particularly high (several-fold that of beech in terms of biomass) and was found to positively influence wild boars population size^38^. At the study site, wild boar females not only experienced masts but were fed additionally. Thus, it would be interesting to study unfed wild boars in mast-failure years, when seed trees completely fail to fructify. We would not, however, expect the absence of mast will lead to a dramatic alteration or even reversal of seasonal rhythms. This is because some of the ruminants investigated^2^, namely red and roe deer, also may take up considerable amounts of energy-rich acorns, in particular in autumn, and in diminishing proportions until January^39,40^. Still, large experimental manipulations of food protein content, or temporal food restriction, only modulated seasonal rhythms in heart rate in red deer, but did not completely reshape them^41^. Hence, it seems that the timing of major peaks and troughs in energy expenditure, the basic seasonal pattern, is a consequence of adaptation to long term average feeding conditions, food selection and gastrointestinal capacity in any given species.

Conceivably, part of the winter increase in energy expenditure may have been due to the costs of thermoregulation. However, the overall impact of T_a_ on HR was relatively small (Table 1). There was a noticeable increase in HR below ~ + 17 °C (Fig. 3a). Surprisingly, the exact lower critical temperature of wild boars seems unknown, but it is at least − 6 °C or below^21^. Thus, an elevated HR that peaked at ~ + 8 °C was probably not related to increased heat production. The slightly elevated HR was associated with higher activity (Fig. 3c), which was generally at a peak during winter (Fig. 1c). Part of this increased activity is due to the onset of mating season in November. Again, this is the opposite of the seasonal activity pattern in other ungulates^2^. Only on very cold days the animals tended to be less active. However, fluctuations of activity where only moderate, which is consistent with the fact that movements of wild boars are least during winters with abundant mast^42^.

Animals in the present study did, however, show clear signs of counteracting cool temperatures. Over the entire range of T_a_ experienced, females lowered subcutaneous T_bsc_ (Fig. 3b). We also observed a slight decrease of T_bip_ with colder T_a_, but it should be noted that intraperitoneal T_bip_ loggers were fixed to the body wall and thus still measured partly shell temperatures. A decrease of subcutaneous T_b_ due to restricted peripheral blood flow seems a common mechanism among ungulates to conserve energy in the cold^2,29^. Arnold et al.^29^ concluded that a decrease in peripheral tissue temperatures served to not only attenuate heat loss but significantly reduced metabolic heat production. Regional heterothermy at night, when T_a_ drops, comes however with a cost since the peripheral tissues have to be rewarmed. Wild boars appeared to largely avoid these costs by basking, whenever solar radiation was sufficient (Fig. 4). Basking is a well-known energy saving mechanism, particularly employed by small mammals rewarming partly passively from torpor e.g.^43–45^. Among large mammals, radiant heat-assisted rewarming has been observed in Alpine ibex that bask in the morning in winter^31^. This allows ibex to increase T_b_ without any additional elevation of HR an hence thermogenesis^31^. Here, we show that wild boars not only rewarm their periphery much faster during basking (Fig. 4a), but even lower energy expenditure as solar radiation increases (Fig. 4b). Using this mechanism, wild boars lowered their mean heart rate by 10% on days with above average temperature differentials between black-bulbs and air. If this translates into a 10% energy saving, it will be quite substantial. It would be interesting to see if wild boars actively seek sunny patches on cold days. Wild boars have further avenues of saving energy, for instance by reducing heat loss via huddling^46,47^. Future studies should assess the quantitative impact of this behaviour for energy budgets in wild boars.

One of the outstanding features of our yearly records of HR was a prominent peak at the beginning of April (Figs. 1a, 2a). This, the months March to May, is exactly the time of year when the majority of females give birth, review in^15^. Wild boar show a remarkable reproductive synchrony that may have evolved to favour polygamy, and among other benefits, facilitates communal nursing, the collective defence against predators, and a greater dilution effect against the individual probability of predation^14^. Here, females showed abrupt changes in heart rate, activity and T_b_ that almost certainly were caused by gestation, retreat to nests, parturition and the onset of lactation (Fig. 2). In swine, the domesticated form of wild boar, it has long been known that there is an abrupt increase of T_b_ at parturition^48^. Also, it was demonstrated before that in other mammals, the birth of litters can be accompanied by sudden changes in physiological variables, e.g., T_b_^49,50^. This finding is interesting in itself, because it means that dates of parturition might be determined in wild, unobserved animals, if they are equipped with suitable data loggers or transmitters. Taken together, we have no doubt that the spring peak in HR, which exceeded all other seasonal changes, was caused by the costs of reproduction, especially increasing energy demands during gestation (Fig. 2a). The latter finding is surprising, because generally the most energy demanding phase in mammals is lactation^51^. Theoretically, peak HR during gestation could be due to an altered relationship between energy expenditure and HR. However, there is no solid physiological basis for this assumption, and empirical data show almost identical ratios of HR/energy-use during gestation and post-partum^52^. Thus, it seems likely that energy-turnover actually decreased during lactation in wild boar females. In domesticated swine food intake does, however, increase during lactation e.g.^53^, but domestication has involved selection for extremely elevated reproductive output. For example, mean litter size in swine is about double that in wild boar^10,54^. In addition, young wild boar are precocial and explore and take up solid food from day 4 to 12 after birth^47^. Hence, it is conceivable that in this species late gestation, not lactation, is the most energy-consuming phase of reproduction in females.

Once more, the seasonal pattern is different from typical northern ungulates, which show a single peak of high energy demand in summer (Fig. 5b). Of course, these species have costs of reproduction as well e.g.^55^ but any increase of HR and energy demands usually coincides with the vegetation related peaks (Fig. 5). Only when females have young very late in the year, outside the main vegetation growth season, the costs of reproduction may become visible in typical grazers^30^.

In wild boar females the birth and peak of energy turnover very early in the year, at least under good environmental conditions, has several reasons. Ultimately it is driven by a selection for an early onset of reproduction. Wild boars can start to breed at a low body weight already in the year of their birth^10,11,16,56^, which selects for parturition early in spring. The broad and highly plastic food choice of wild boars may have been a prerequisite for early sexual maturity. Proximately, reproductive success strongly depends on female condition e.g.^57^ but also on lactation. Although the precocial juveniles start to take in solid food early, females still lactate for 3–4 months^46^. Thus, lactation is typically terminated before summer and hence before energy turnover comes to a seasonal low in June–August (Fig. 1a). Expectedly, this pattern should be pronounced in hot and dry summers. In our study females were supplementary fed throughout the year and did not experience a severe food reduction during summer. In free-ranging wild boar populations, however, rooting is the main foraging mode in many areas, and is particularly important in summer when other food sources are scarce^15^. A dry soil in hot and dry summers will particularly impede digging up insects, grubs and worms and thus lower food availability. In fact, both summers in the study years 2017 and 2018 were among the four warmest recorded ever, and 2018 was additionally characterised by a severe drought^58,59^.

Theoretically, energy expenditure might be quite different in male wild boars. For instance, adult males may lose body mass during the winter rut even when food supply is high. At the same time, females may even gain body mass, indicating clear differences between sexes in energy intake or -expenditure during winter^13^. Hence, we would predict that energy expenditure among males is higher during the rut leading to an even higher fall/winter peak in heart rate. Also, we assume that its spring peak is due to late gestation and parturition and thus missing in males. We have, however, no reason to suspect an entirely different seasonal shape of energy expenditure of the two sexes.

## Conclusions

In wild boars a strong seasonal rhythm in heart rate, a proxy of energy expenditure, is not primarily caused by costs for thermoregulation. A spring peak in energy expenditure is driven by the costs of reproduction in females, a secondary peak in autumn/winter coincided with the availability of mast and the mating season. The shape of seasonal changes seems governed by selection for reproduction early in the year, and differs from other north-temperate ungulates, likely due to the broad food spectrum of wild boars. Thus, high food availability may completely offset the energetic costs of seasonal climates, and winter is not always the energetically most challenging season. In the face of the ongoing global warming, extremely warm and dry summers may in fact increasingly become the yearly energetic bottleneck for wild boars.

## Material and methods

### Ethical statement

The present study was discussed and approved by the ethics and animals’ welfare committee of the University of Veterinary Medicine, Vienna, Austria, in accordance with good scientific practice and national legislation (GZ: BMWFW-68.205/0151-WF/V/3b/2016 and GZ: BMWFW-68.205/0224-WF/V/3b/2016). All methods were carried out in accordance with relevant guidelines and regulations. We confirm that the study was carried out in compliance with the ARRIVE guidelines. No plants or plant parts were used in this study.

### Animals and study area

The study animals were kept in an outdoor enclosure (~ 55 ha, for details see “Supplementary Material”). The study enclosure was covered with a deciduous forest, mainly Turkey oak (*Quercus cerris*) and pubescent oak (*Quercus pubescens*) and included only few meadow patches. For the present study ten adult females, were used. We concentrated on females only because the live capture and handling of males are hampered by the large size and ferocity of boars. Also, due to competition and high levels of aggression between males during rut, the stocking of the enclosure was strongly female biased. During the study period (12/2016–01/2019), the animal density was ~ 1 adult female/ha plus up to 20 males (total) of different ages. Due to this relatively high density, animals were supplemented with 1–1.5 kg corn/individual once a day (at 2:00–14:00 h) at two feeding areas, each ~ 40 × 20 m. The enclosure was part of a game reserve, which was enclosed by 2.5 m high, solid, non-transparent fencing and was closed for the public. Thus, the study site provided an environment without disturbances due to hikers, bikers or straying dogs. There were no battue hunts or other disturbances due to hunting or forest management activities during the study period in the enclosure.

Animals were trapped once a year in autumn within the feeding sites to collect data on reproductive success and body condition of females and to separate some of them for implantation/explantation of loggers. While feeding, we closed the access gates and released the boars one by one trough a wooden corridor back into the enclosure. While in the wooden corridor we recorded the body mass of each individual (Gallagher SmartScale® 500, Groningen, Netherlands). Due to management reasons the juveniles (born in spring) were removed from the enclosure during this procedure.

### Implantation of temperature and heart rate loggers

We implanted a heart rate logger (DST centi-HRT, Star-Oddi, Gardabaer, Iceland) and two custom-built temperature loggers in each of ten female wild boars in October/November 2016 and 2017 (age 5 and 6 years). All details about surgery techniques and anaesthesia protocols are provided in the “Supplementary Material”. Explantations were carried out approximately one year after implantations. The last explanation was carried out in January 2019. One female was implanted in two consecutive years. Mean body mass at date of implantation for all females was 71.8 ± 15.5 kg.

The heart rate logger was adjusted to record data at a time interval of 12 min to cover one year of data recording. To remove outliers, all initial data from these recorders were subjected to a running median over five consecutive values. The HR recorder was positioned subcutaneously, in proximity to the heart on the lateral rib cage, behind the moving area of the elbow, to avoid rubbing, or inserted and tethered into the ventral subperitoneal space caudal of the xiphoid process of the sternum.

The self-built temperature loggers were covered with inert surgical wax and had a weight of ~ 8 g. Time interval of recording was 4 min, the accuracy 0.01 °C. One of the two temperature loggers had an especially flat shape (3.4 × 1.9 × 0.5 cm) to fit smoothly into the subcutaneous neck region. The second temperature logger was placed into the intraperitoneal cavity, tethered at the *Linea alba* (diameter = 2.1 cm, height = 1.2 cm). For details on surgery, see “Supplement”.

We collected and evaluated a mean of 227.45 ± 160.69 days of heart rate recording per individual (SD, n = 11: 33 days, 58 days, 79 days, 89 days, 143 days, 189 days, 272 days, 345 days, 412 days, 421 days, 461 days), and a mean of 382.00 ± 100.17 days (SD), of subcutaneous logger recording per individual (n = 8: 143 days, 363 days, 411 days, 414 days, 419 days, 421 days, 424 days, 461 days). From the loggers implanted in the abdominal cavity we collected 338.71 ± 117.01 days (SD) per individual (n = 10: 140 days, 143 days, 363 days, 364 days, 411 days, 419 days, 421 days, 421 days, 424 days, 461 days). The hourly means of monitored heart rates of each animal over the course of the year are shown in Supplementary Fig. S1.

### Activity data

To record the activity of animals, a telemetry system (Smartbow System, Zoetis, New Jersey, USA) was installed around the two neighbouring feeding areas and two close water ponds in the enclosure. The system consisted of a central solar power and computing station and ten receivers located at the height of 2–3 m. Part of the system were ear-tags (34 g; 52 mm × 36 mm × 17 mm, for details see “Supplementary Material”). The accelerometer (located inside ear-tags) measured triaxial acceleration (x, y, z). As an estimate of locomotor activity (ACT), we computed the total acceleration vector from sqrt (x^2^ + y^2^ + z^2^).

### Climate and mast

The study site in Eastern Austria (altitude 130 m) is generally characterised by a Pannonian climate. According to long-term climate records, the mean annual temperature is 10 °C in combination with a mean precipitation of 600–700 mm and 1898 h of sunshine per year (ZAMG, 1971–2000).

We recorded ambient temperature (T_a_) and black bulb temperature (T_ab_) at 2 m height directly at the study site (Vantage Pro 2 with black bulb extension, Davis Instruments, Hayward, USA).

To assess the extent of the acorn mast, each autumn seven nets, 4 × 4 m, were set up to collect acorns at random locations. The nets were regularly emptied between Sept. and Nov. each year, and the collected acorns were dried and weighed. In the autumns prior to the study (2016) and during both full study years (2017/2018) there was seeding of at least part of the oaks. Over ~ 90 days in each autumn we collected 52.4 g/m^2^, 134.8 g/m^2^, and 37.5 g/m^2^ acorn in 2016, 2017, and 2018, respectively. Thus, 2017 was a full mast year but there were acorns available in autumn throughout the study period.

### Data analysis

To facilitate handling of data and to reduce autocorrelation we compiled and evaluated hourly means for all data, i.e., heart rates (HR; see Suppl. Fig. S1), intraperitoneal and subcutaneous body temperature (T_bip_ and T_bsc_, respectively) and activity (ACT), as well as ambient air temperature (T_a_) and black-bulb temperature (T_ab_). We further tested for effects of day of year (DOY) and hour of day (HOUR). We did not assess the influence of environmental conditions in different years, because due to logger-failures and thus scarcity of heart rates, all data were pooled for different years (with similarly warm conditions and food available year-round). Also, we did not further evaluate daily rhythms, because animals were always fed in the early afternoon, which may have influenced their timing.

We investigated the effects of season (DOY), hour of day (HOUR), and T_a_ on the response variables HR, T_bip_, T_bsc,_ and ACT. We additionally used T_bip_, T_bsc,_ and ACT as predictors for HR. As many of the relationships between these were non-linear, we used general additive mixed models (GAMMs), as implemented in package mgcv^60^ in R^61^. This function fits non-linear splines to the data, which are penalized for their “wiggliness”, i.e., the number of turning points in the fit. Because the data were repeated measurements, we calculated for all response variables mixed models with an intercept for each animal ID as a random factor (using s (ID, bs = ”re”)). Hence, these mixed models allowed for differences in the mean level of heart rates, temperatures and activities, between individuals. All residuals of models were approximately normally distributed, as inspected by normal quantile–quantile plots. Hourly means of the response variables contained various degrees of autocorrelation. This was corrected by including autoregressive order 1 (AR1) error models in GAMM-functions, which successfully reduced the autocorrelation at lag 1 to nonsignificant levels. This was confirmed by comparing the autocorrelation function of model residuals (ACF) before and after their correction. To illustrate the effects of independent variables, we show population-level predictions from GAMMs. These graphs contain rug plots to illustrate the distribution of independent variables. Because these plots were too dense for all original data (resulting in black bars), we show uniform random samples (n = 1000) from each independent predictor variable.

Because hourly mean data consisted of ~ 117,000 observations we used the mgcv function “bam”, which uses numerical methods designed for large datasets. To fit non-linear functions to predictors, we used the default thin plate splines. Only the cyclic variables DOY and HOUR were modelled using cubic cyclic splines, which are guaranteed to have identical start- and endpoints (e.g., at Jan 1 and Dec 31). GAMMs were always fitted using method REML. As T_bip_ and T_bsc_ were only moderately correlated (r = 0.30), both were entered simultaneously as independent variables in the model on heart rate.

We did not use partial regression plots from multiple regressions that included activity. This is because activity could only be recorded partly, in the vicinity of telemetry receivers. Thus, models that include ACT as well as all other predictors simultaneously, were restricted to ~ 7% of the data. However, we still used a full multiple regression model HR for the purpose of assessing relative variable importance (of DOY, HOUR, Ta, T_bip,_ T_bsc,_ and ACT). F-values from this model provide an indication of the importance of different predictors.

To model a possible role of solar radiation and basking we computed the difference between T_ab_ and T_a_, called T_diff_, which represents an index of radiation. We used again GAMMs to test if T_diff_ would affect T_bip_, T_bsc_ and HR after adjusting for effects of T_a_, hour of day, and the random factor animal ID.

For a comparison of species we also computed monthly means and SEMs of HR in wild boars, and created a graph of seasonal time courses in other ungulates as published in Arnold^2^ that were kindly provided by the author. If not stated otherwise we provide means ± SEM.

## Supplementary Information

Supplementary Information.

## Data Availability

Data will be made available on Phaidra (https://www.vetmeduni.ac.at/en/bibliothek/infoservice/phaidra/).
