# Supplementary material for: Atypical for northern ungulates, energy metabolism is lowest during summer in female wild boars (Sus scrofa)
Source: Sci Rep. 2021 Sep 15;11:18310. doi: 10.1038/s41598-021-97825-z (PMC8443605; doi:10.1038/s41598-021-97825-z)
Supplement: Supplementary file 1 — Supplementary Information. [file 41598_2021_97825_MOESM1_ESM.pdf]

Supplementary Material

Atypical for northern ungulates, energy metabolism is lowest during summer in female wild boar (*Sus scrofa*)

Thomas Ruf[1*], Sebastian G. Vetter[1, 2], Johanna Painer[1], Gabrielle Stalder[1], Claudia Bieber[1]

[1] Research Institute of Wildlife Ecology
Department of Interdisciplinary Life Sciences
University of Veterinary Medicine, Vienna, Austria
Savoyenstrasse 1, A-1160 Vienna, Austria

[2] Present Address:
Institute of Animal Welfare Science
Department for Farm Animals and Veterinary Public Health
University of Veterinary Medicine, Vienna, Austria
Veterinärplatz 1, A-1210 Vienna, Austria

***Corresponding author:** Thomas Ruf[1], thomas.ruf@vetmeduni.ac.at

Supplementary Material

Animals and surgery

118 female wild boar piglets were purchased in to our enclosure at the age of ~6 months in autumn 2011. The transport, animal marking, and the keeping conditions fulfilled ethical and legal requirements and are described in detail in Vetter et al. (2016).

For implantation, animals were led through wooden corridors and captured in wooden boxes (1.2 x 0.7 x 1.1m) at the feeding sites. In the box they were anaesthetized with an injection with a pole-syringe in the neck-muscle behind the ear base (Jab Stick CATSR, Dan-Inject, Borkop, Denmark), using a combination of 3 mg/kg tiletamine-zolazepam (Zoletil 100®, Virbac Austria GmbH, Vienna, Austria), 0.06 mg/kg medetomidine (Medetomidine 20mg/mL, magistral formulation, Vienna, Austria), 0.15 mg/kg Butorphanol (Alvegesic®, Alvetra u. Werfft Animal Nutrition GmbH, Vienna, Austria) IM. Drugs were calculated to the exact body mass.

To protect the eyes during surgery eye ointment (Vit-A-Vision®, Omnivision GmbH, Puchheim, Germany) was applied and the eyes were covered with a blindfold. Rectal temperature was monitored closely using a digital thermometer. Values of oxygen saturation of the blood (SpO_2), heart rate, respiratory rate, end-tidal CO_2, body temperature, blood pressure were continuously measured and monitored during anaesthesia (Masimo Root®-Root with Noninvasive Blood Pressure and Temperature Monitoring; Radical-7®; Phasein ISA™; Masimo Corporation, CA, USA). Anaesthetic depth was assessed by testing the palpebral, perineal and pedal reflex. All individuals were intubated, received oxygen and isotonic infusions. To shorten the time in anaesthesia, the implantations of the loggers were carried out by two veterinarians in parallel. Areas of implantation were shaved, aseptically prepared and covered by surgical drapes.

For the subcutaneous loggers a subcutaneous skin pocket was built to position the logger. The incision was closed with an intracutaneous and simple interrupted suture (Surgicryl PGA USP 0, SMI AG, Hünningen, Belgium). The intraperitoneal cavity was opened by a 2 cm incision in the *linea alba*, to position one logger for recording of the core body temperature. To facilitate explanation, this logger was fixed to the internal abdominal wall at the *linea alba*.

The abdominal wall (Surgicryl PGA USP 1, SMI AG, Hünningen, Belgium), subcutis and skin (Surgicryl PGA USP 0, SMI AG, Hünningen, Belgium) were closed with simple interrupted and intracutaneous suture respectively. All animals were administered pre-surgically 0,4mg/kg Meloxicam (Boehringer Ingelheim Vetmedica GmbH, Ingelheim am Rhein; Germany) IM. After surgery, the medetomidine component was reversed with the administration of IM atipamezole (Narco Stop, Richter Pharma AG, Austria), dosed at 5 mg for each mg of medetomidine given. The animals were monitored until recovery (approximately 2–4 hours), before released into their respective enclosure.

Activity recording

Accelerometers we built into ear-tag and these data were sent every 4 s (when the animal was active) and every 16 s (when the wild boar was inactive) to the receivers. According to capture-recapture schedules once a year and battery capacity, acceleration data were recorded at 1 Hz. Temperature data were recorded once per transmission (i.e., every 4 s or 16 s, respectively). The transmission was achieved through a wireless local area network (WLAN). Data were not stored in the ear tag if the animals were outside the receiving area. Receiving distance was 300 m in open areas but decreased within forests and shrubby areas due to the barrier of fluid water. In total, the telemetry system (10 receivers) covered an area of ~4 ha. Data received were send via mobile internet to a Smartbow-server, where they were stored and then downloaded for evaluation.

Vetter, S.G., Brandstätter, C., Macheiner, M., Suchentrunk, F., Gerritsmann, H. & Bieber, C. (2016). Shy is sometimes better: personality and juvenile body mass affect adult reproductive success in wild boars, *Sus scrofa*. *Animal Behaviour, 115*, 193-205. https://doi.org/10.1016/j.anbehav.2016.03.026

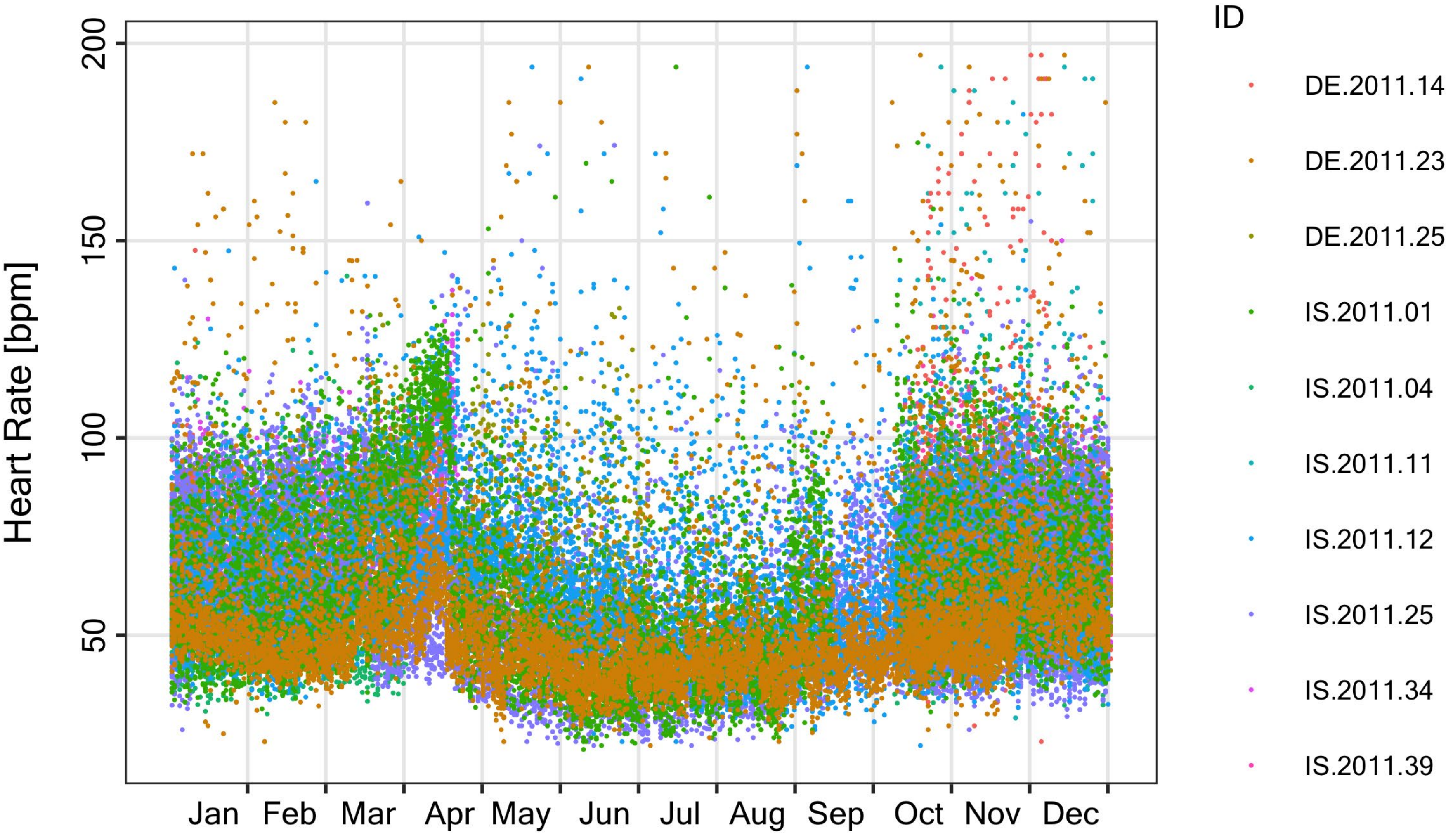

Supplementary Fig. S1. Heart rates of 10 adult, female wild boar over the course of one year. Hourly means including both rest and activity as subjected to GAMM analysis.
